# Characteristics of Patients Treated for Orbital Infections: A Retrospective Study at a Tertiary Care Hospital

**DOI:** 10.7759/cureus.79004

**Published:** 2025-02-14

**Authors:** Saleh Khurshied, Hira G Shah, Wafa Abdul Malik, Aunaza Maqbool, Khadija Hussain, Muhammad A Zahid

**Affiliations:** 1 Otolaryngology - Head and Neck Surgery, Pakistan Institute of Medical Sciences, Islamabad, PAK; 2 Ophthalmology, Al-Shifa Trust Eye Hospital, Rawalpindi, PAK; 3 Ophthalmology, Monash Health, Clayton, AUS

**Keywords:** cellulitis orbit sinusitis antibiotics, conservative management, orbital cellulits, sinusitis, successful treatment outcome, treatment choices

## Abstract

Introduction

The orbital tissues of the eye can be affected by infections, a serious clinical illness that can have potentially fatal consequences. The study aimed to identify the demographic data, the categories of orbital infections based on Chandler's classification, and the underlying causes of orbital infections to ascertain the characteristics of patients admitted with orbital infections. We also looked at the management practices and their results.

Materials and methods

The Department of ENT - Head and Neck Surgery at the Pakistan Institute of Medical Sciences Hospital in Islamabad was the chosen site of this retrospective study. A total of 63 patients were included in the study. Data including patients' age, gender, type of orbital infection, inciting cause, management carried out, and treatment success were extracted from their discharge forms between January 2022 and December 2024. Following statistical analysis, the information collected was presented as tables and figures.

Results

Out of the total, 40 (63.49%) patients were female. The median age of patients was 34.5 years. Most patients were diagnosed with subperiosteal abscesses (33, 52.38%). The most common cause was sinusitis, which was present in 41 (65.07%) patients. Out of the total, 47 (74.60%) patients were successfully managed conservatively using broad-spectrum antibiotics and symptomatic treatment, and 12 (19.05%) underwent surgical intervention to drain the abscess.

Conclusions

The orbital infections were most common in middle-aged female patients, with subperiosteal abscess being the most common type. Sinusitis was the most common cause. Most patients were managed successfully with intravenous antibiotics, with few needing surgical intervention.

## Introduction

Life-threatening problems can result from the infections of orbital tissues, a serious clinical illness that affects the orbital tissues and adnexal structures of the eye. Attached to the orbital base margins, the orbital septum is a thin, fibrous structure that divides the inside orbital tissues from the palpebral soft tissues. Orbital cellulitis and preseptal cellulitis are infections of the tissues behind and in front of the orbital septum, respectively [1-3]. Regional invasion of pathogenic agents from the sinuses, nasopharynx, and surrounding tissues; trauma; surgery; blood-borne infections; or dental infections can all result in orbital infections [4,5].

The main clinical criteria for diagnosing orbital cellulitis are the patient's physical examination and presenting signs and symptoms. Ophthalmoplegia, pain in eye movements, and/or proptosis are the three main characteristics of orbital cellulitis. Imaging techniques, including CT and MRI, help confirm the diagnosis of orbital infections [6]. *Staphylococcus aureus* and *Streptococcus* are the most common organisms causing orbital cellulitis [7].

The Chandler classification is the most often used to distinguish between various kinds of orbital cellulitis. According to the system, there are five stages [8]. In Stage I, orbital cellulitis is preseptal and presents with edema and inflammation anterior to the orbital septum. In Stage II, the edema and inflammation extend past the orbital septum. A subperiosteal abscess under the periosteum of the lamina papyracea, an orbital abscess in the orbit's intraconal region, and cavernous sinus thrombosis following the infection spreading through the superior ophthalmic veins are the third, fourth, and fifth stages, respectively.

Although intravenous antibiotics are the mainstay of treatment for orbital cellulitis, surgical drainage of abscesses may be required to relieve pressure and avoid complications or failure to respond to medical treatment [9,10].

Through this study, we aim to determine the characteristics of the patients admitted to the ENT ward with orbital infections. To find the most common type of orbital infection based on Chandler’s classification and the inciting cause of the orbital infection. To see the management done in our hospital and its outcomes.

This research aimed to provide the basic details regarding orbital infections. From determining the common cause of orbital infections to providing effective care, we provided the fundamental framework for managing this serious disease by looking at the outcomes and analyzing the results retrospectively.

## Materials and methods

After receiving formal written consent from the department head to use the discharge data from the department records from January 2022 to December 2024, this retrospective descriptive observational study was conducted at the Department of ENT - Head and Neck Surgery, Pakistan Institute of Medical Sciences (PIMS) Hospital, Islamabad, after taking formal written consent from the department head to use departmental data for research. PIMS is a tertiary care government hospital that has designated 46 beds for ENT and ophthalmology patients. A total of 63 patients during the study period fulfilled the inclusion criteria. Demographic information was gathered, including age, gender, diagnosis according to Chandler's classification [8], medical or surgical treatment done at the hospital, and outcome upon discharge. Patients with inadequate medical records who were not admitted within the aforementioned time frame, pregnant females, and patients with multiple comorbidities were excluded from the study. In contrast, those who were admitted with a diagnosis of orbital infection (Chandler stages 1-5, including preseptal cellulitis, orbital cellulitis, subperiosteal abscess, orbital abscess, and cavernous sinus thrombosis, respectively) between the aforementioned dates were included.

The collected data was entered and statistically analyzed using SPSS Statistics version 25.0 (IBM Corp. Released 2017. IBM SPSS Statistics for Windows, Version 25.0. Armonk, NY: IBM Corp.). Numbers and percentages were calculated for different variables. Descriptive data was presented as frequencies and percentages displayed as tables and charts. The primary variables under study were type of infection, cause, and management done, while age, gender, and treatment outcome were secondary variables.

Information that could be used to identify patients was concealed during data collection to protect patient confidentiality. There was no direct communication with any of the patients or their relatives.

## Results

**Figure 1:**
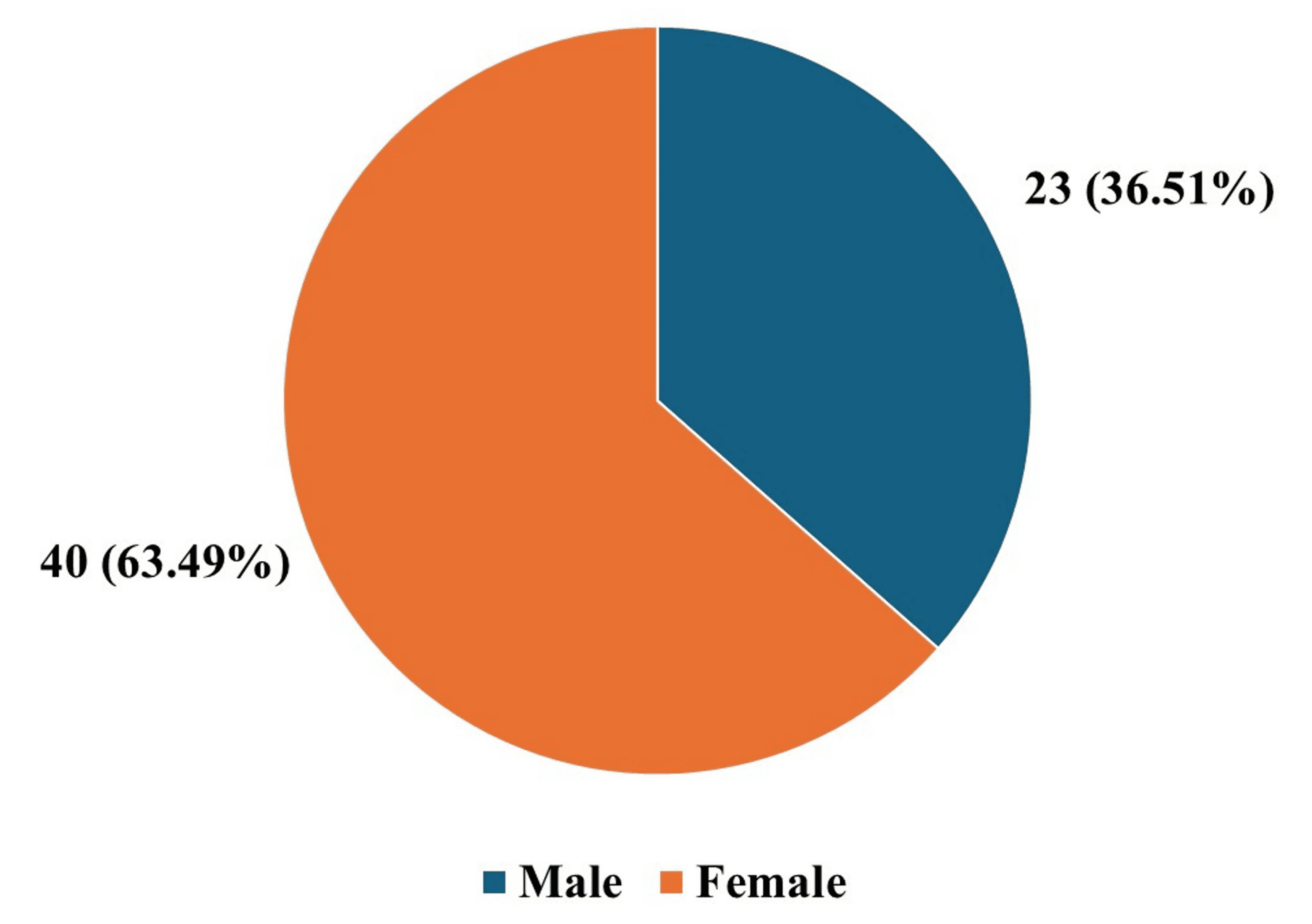
Gender of patients included in the study Number of admitted male and female patients over the two years of study duration (January 2022 to December 2024) along with the percentage proportion of total patients for each gender in brackets.

**Figure 2:**
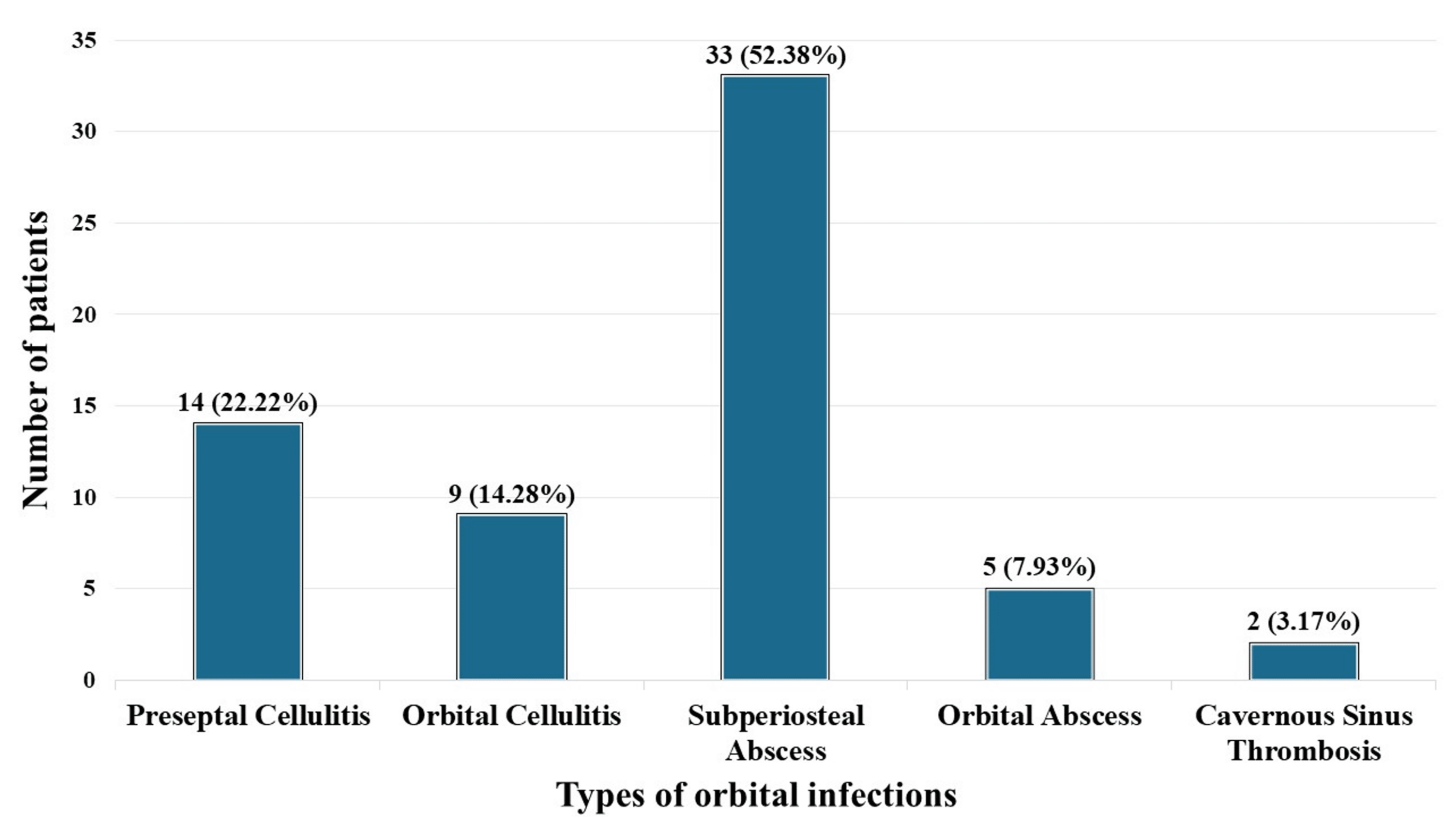
Number of patients with different types of orbital infections y-axis (number of patients): The number of patients corresponding to the disease. x-axis (types of orbital infections): The different types of orbital infections, as per Chandler's classification.

**Figure 3:**
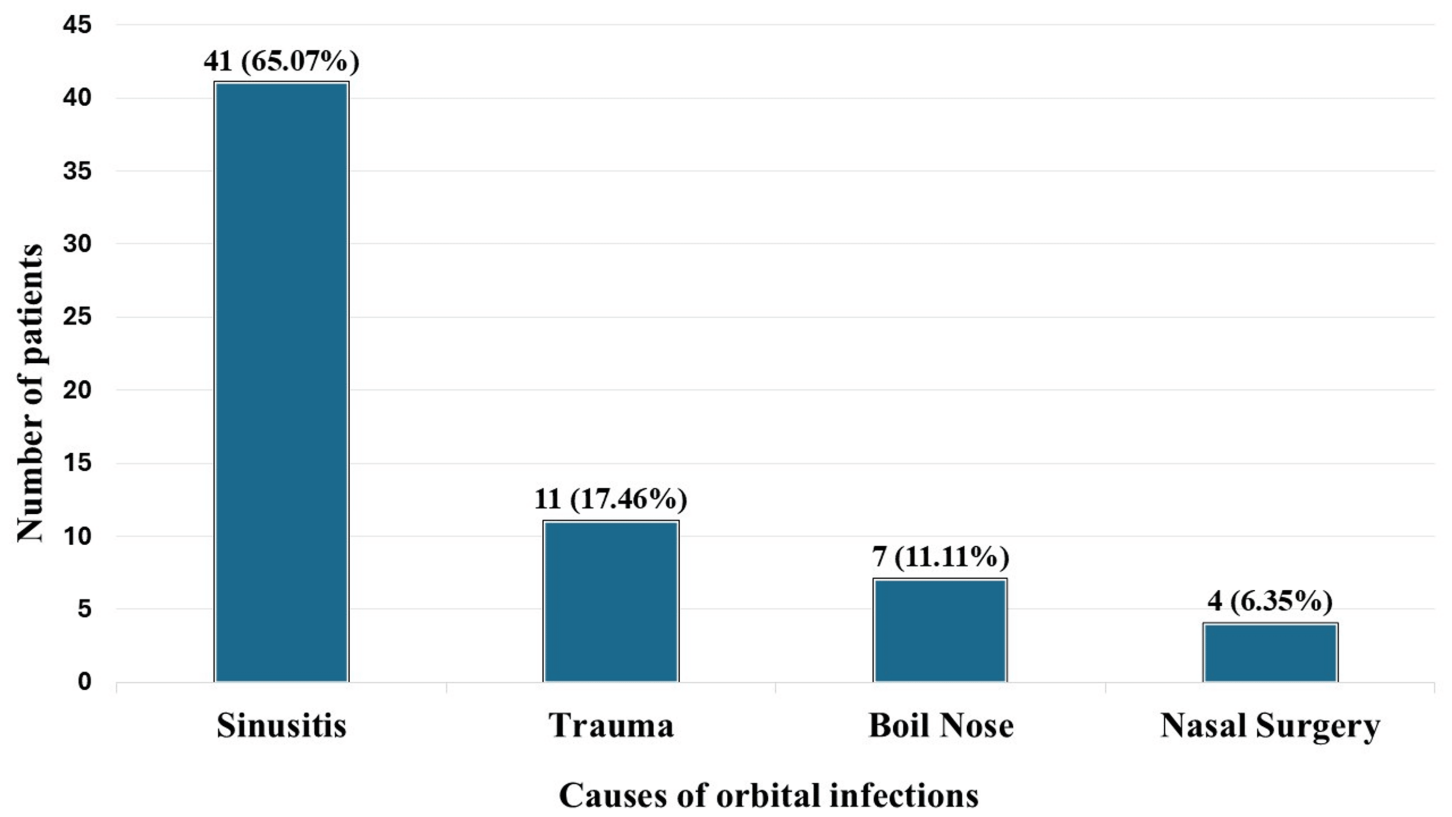
Predisposing causes of orbital infections y-axis (number of patients): The number of patients corresponding to the cause of disease. x-axis (causes of orbital infections): The different causes of orbital infections in the patients included in the study.

Sixty-three patients were admitted with orbital infection during the study period. Of the total, 23 (36.51%) patients were male, and 40 (63.49%) were female, as shown in Figure 1. The patients were between the ages of 5 and 68, with a median age of 34.5 years. Most patients (21, 33.33%) were between 30 and 40 years old, as detailed in Table 1. A subperiosteal abscess was identified in 33 (52.38%) of the patients, followed by preseptal cellulitis in 14 (22.22%), orbital cellulitis in nine (14.28%), orbital abscess in five (7.93%), and cavernous sinus thrombosis in two (3.17%), as shown in Figure 2. Sinusitis accounted for 41 (65.07%) of the cases, with trauma as an inciting cause in 11 (17.46%), boil nose in seven (11.11%), and nasal surgery in four (6.35%), as detailed in Figure 3.

**Table 1:** Age range of patients included in the study Age range (years): The range of ages, in years, of the patients admitted with orbital infections during the study period. Number of patients (%): The number of patients in that age group and the percentage proportion in that age group in brackets.

Age range (years)	Number of patients (%)
0-10	11 (17.46)
11-20	4 (6.35)
21-30	7 (11.11)
31-40	21 (33.33)
41-50	13 (20.63)
51-60	5 (7.94)
61-70	2 (3.17)

**Figure 4:**
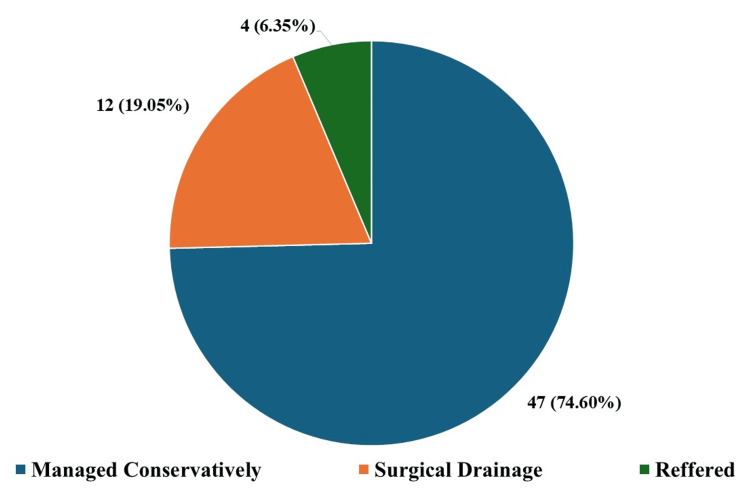
Types of management done to patients Different management plans used to manage patients over the past two years (January 2022 to December 2024) along with the percentage proportion of total patients for each type of management plan in brackets.

As shown in Figure 4, 47 patients (74.60%) were effectively managed medically with broad-spectrum antibiotics and symptomatic treatment (standard analgesics and anti-inflammatories), 12 patients (19.05%) had surgery to drain the abscess, and four patients (6.35%) were sent to another specialty for management (eye, neurology, or neurosurgery).

## Discussion

Orbital cellulitis is a serious eye condition that necessitates immediate hospitalization, as it can lead to blindness, vision loss, and even death [10]. This warrants urgent medical and/or surgical interventions by ophthalmologists and otolaryngologists [11]. Most patients (63.49%) in our research were female, but 61% of cases observed by Santos et al. [12] were male, which may be due to geographical and racial differences. As in our study, the most common cause of orbital cellulitis was sinusitis in Hayat et al.'s study [13]. Similarly, the most prevalent predisposing factor for orbital cellulitis was sinus disease, which was present in 39.4% of patients, followed by trauma in 19.7% [7]. A very high incidence of sinusitis (91.2% and 94.7%, respectively) was observed in studies conducted on children in Australia and Canada [14,15]. Mohd-Ilham et al. [16] discovered sinus infection was the most frequent cause of orbital cellulitis. Hayat et al. [13] found that 21% of patients required surgery, while Mahalingam-Dhingra et al. [17] found that 12.4% required surgery. These outcomes closely matched our findings, which showed that 19.05% of cases in our study required surgical intervention. In a similar vein, Fanella et al. [15] reported that 21.1% of their patients had surgery. The average age of these patients in the study [7] was 25.7 years (one month to 85 years), which was more in line with our findings. Most orbital collections were subperiosteal, as was the case in our study, according to Yen and Yen et al. [18]. Mishra et al. [19] found that 45% of patients with orbital cellulitis had predisposing sinusitis, and 70% of their patients were male, and these findings were consistent with ours. Murphy et al. [20] treated all their patients with intravenous antibiotics, which was closer to the management plan we found in our hospital.

One advantage of this study is that it offers the first insights into the characteristics of orbital infections in Pakistan. Another benefit is the research's retrospective character, which provides future researchers with an explanation of the data, management strategies, and results. Among the study's limitations were the limited sample size and the fact that the data was only collected from one institution for a brief period of time. The possibility of documenting inaccuracies in medical records is another drawback. We recommend additional research with a big sample size from multiple hospitals comparing different attributes of orbital infections.

## Conclusions

The majority of patients with orbital infections were early middle-aged women. A subperiosteal abscess was the most prevalent kind of orbital infection. The most prevalent known predisposing cause for orbital infection development was sinusitis. Most patients were well treated with intravenous antibiotics, and a small number required surgery.
